# Supplementary material for: Asynchronous magnetic resonance elastography: Shear wave speed reconstruction using noise correlation of incoherent waves
Source: Magn Reson Med. 2022 Oct 27;89(3):990–1001. doi: 10.1002/mrm.29502 (PMC9792433; doi:10.1002/mrm.29502)
Supplement: Supplementary file 1 — DATA S1 MATLAB code used to generate the numerical simulations of Figure 1 [file MRM-89-990-s002.zip › k-Wave/helpfiles/makeMultiBowl.html]

makeMultiBowl :: Functions (k-Wave)


# makeMultiBowl

Create a binary map of multiple bowls within a 3D grid.

## Syntax

```
[bowls, bowl_labelled] = makeBowl(grid_size, bowl_pos, radius, diameter, focus_pos)
[bowls, bowl_labelled] = makeBowl(grid_size, bowl_pos, radius, diameter, focus_pos, ...)
```

## Description

`makeMultiBowl` creates a binary map of multiple bowls within a three-dimensional grid using `makeBowl`. The position of the bowls is denoted by 1's in the the matrix with 0's elsewhere. A labelled matrix can also be returned, where the position of the first bowl is denoted by 1's, the position of the second bowl by 2's, and so on.

## Examples

```
% define grid parameters
x_size              = 300e-3;
Nx                  = 256;
dx                  = x_size / Nx;
grid_size           = [Nx, Nx, Nx];

% create a Cartesian sphere with the x, y, z positions of the bowls
sphere_radius       = 125e-3;
num_bowls           = 64;
bowl_pos            = makeCartSphere(sphere_radius, num_bowls, [1, 1, 1] * x_size / 2).';

% convert the Cartesian bowl positions to grid points
bowl_pos            = round(bowl_pos/dx);

% define element parameters
radius              = round(x_size / (2 * dx));
diameter            = 21;
focus_pos           = [1, 1, 1] * Nx/2;

% create bowls
makeMultiBowl(grid_size, bowl_pos, radius, diameter, focus_pos, 'Plot', true);
```

## Inputs

|  |  |
| --- | --- |
| `grid_size` | size of the 3D grid given as a three element vector [Nx, Ny, Nz] [grid points] |
| `bowl_pos` | centre of the rear surface of each bowl given as matrix of dimensions N x 3, with each row specifying the centre for each bowl as a three element vector [bx, by, bz] [grid points] |
| `radius` | radius of curvature of each bowl given as either a single number (if the bowls have the same radius), or an N-element vector containing the radius for each bowl [grid points] |
| `diameter` | aperture diameter of each bowl given as either a single number (if the bowls have the same diameter), or an N-element vector containing the diameter for each bowl [grid points] |
| `focus_pos` | any point on the beam axis of the bowl given as either a three element vector (if the bowls have the same focus\_pos), or as a matrix of dimensions N x 3, with each row specifying the focus\_pos for each bowl given as a three element vector [fx, fy, fz] [grid points] |

## Optional Inputs

Optional 'string', value pairs that may be used to modify the default computational settings.

| Input | Valid Settings | Default | Description |
| --- | --- | --- | --- |
| `'Binary'` | *(Boolean scalar)* | `false` | Boolean controlling whether the bowl map is returned as a double precision matrix (false) or a logical matrix (true). |
| `'Plot'` | *(Boolean scalar)* | `false` | Boolean controlling whether the bowl array is plotted using `voxelPlot`. |
| `'RemoveOverlap'` | *(Boolean scalar)* | `false` | Boolean controlling whether overlapped grid points within each bowl (not between bowls) are removed. |

## Outputs

|  |  |
| --- | --- |
| `bowls` | 3D binary map of a bowls |
| `bowls_labelled` | 3D labelled matrix of bowls |

## See Also

`makeBowl`, `makeMultiArc`
